# Spring Ephemerals Adapt to Extremely High Light Conditions via an Unusual Stabilization of Photosystem II

**DOI:** 10.3389/fpls.2015.01189

**Published:** 2016-01-06

**Authors:** Wenfeng Tu, Yang Li, Wu Liu, Lishuan Wu, Xiaoyan Xie, Yuanming Zhang, Christian Wilhelm, Chunhong Yang

**Affiliations:** ^1^Key Laboratory of Photobiology, Institute of Botany, Chinese Academy of SciencesBeijing, China; ^2^Key Laboratory of Biogeography and Bioresource, Xinjiang Institute of Ecology and Geography, Chinese Academy of SciencesUrumqi, China; ^3^Institute of Biology, Department of Plant Physiology, University of LeipzigLeipzig, Germany

**Keywords:** D1 protein turnover, electron transport, light stress, photoinhibition, photosystem activity, spring ephemeral

## Abstract

Ephemerals, widely distributed in the Gobi desert, have developed significant characteristics to sustain high photosynthetic efficiency under high light (HL) conditions. Since the light reaction is the basis for photosynthetic conversion of solar energy to chemical energy, the photosynthetic performances in thylakoid membrane of the spring ephemerals in response to HL were studied. Three plant species, namely two C_3_ spring ephemeral species of Cruciferae: *Arabidopsis pumila* (*A. pumila*) and *Sisymbrium altissimum* (*S. altissimum*), and the model plant *Arabidopsis thaliana (A. thaliana)* were chosen for the study. The ephemeral *A. pumila*, which is genetically close to *A. thaliana* and ecologically in the same habitat as *S. altissimum*, was used to avoid complications arising from the superficial differences resulted from comparing plants from two extremely contrasting ecological groups. The findings manifested that the ephemerals showed significantly enhanced activities of photosystem (PS) II under HL conditions, while the activities of PSII in *A. thaliana* were markedly decreased under the same conditions. Detailed analyses of the electron transport processes revealed that the increased plastoquinone pool oxidization, together with the enhanced PSI activities, ensured a lowered excitation pressure to PSII of both ephemerals, and thus facilitated the photosynthetic control to avoid photodamage to PSII. The analysis of the reaction centers of the PSs, both in terms of D1 protein turnover kinetics and the long-term adaptation, revealed that the unusually stable PSs structure provided the basis for the ephemerals to carry out high photosynthetic performances. It is proposed that the characteristic photosynthetic performances of ephemerals were resulted from effects of the long-term adaptation to the harsh environments.

## Introduction

Ephemerals are widely distributed in the Gobi desert and play important roles in maintaining and restoring the desert ecosystems (Wang et al., 2003). To survive the harsh environmental conditions such as extremely high irradiance and high temperature, ephemerals in the desert areas have evolved many distinctive adaptation mechanisms. Phenomenologically, ephemerals are able to accomplish their life cycle quickly under strong light conditions (Ehleringer et al., 1979; Yuan et al., 2009). Ephemerals possess high photosynthetic activity under strong light conditions because of their extremely high photosynthetic light saturation point that enables their photosynthetic apparatus to sustain efficient photosynthesis without suffering from any photodamage even under full sunlight (Ehleringer, 1983; Yuan et al., 2009). Indeed, the electron transport and photosynthetic CO_2_ uptake in ephemerals are not saturated at light intensities under 2000 μmol photons m^−2^ s^−1^ (Ehleringer, 1983; Tu et al., 2012). Accordingly, ephemerals show only low NPQ values and no photoinhibition, because the photochemical efficiency is high at any light intensity within the scope studied (Eppel et al., 2014). However, the mechanism of these unusual photosynthetic regulations have not been analyzed in detail so far. In order to get a better understanding of ephemerals’ photosynthesis, it should be known which components of the photosynthetic primary reactions are modified.

Under strong light conditions when the energy excitation exceeds the capacity of carboxylation, plants have to develop mechanisms preventing an over-reduction of the photosynthetic electron transport chain to prevent the generation of ROS and the inactivation of the photosynthetic functions (Demmig-Adams and Adams, 2000; Murata et al., 2007; Vass, 2012). Recent research has documented highly flexible and dynamic changes in the thylakoid membranes and various photoprotective mechanisms, including the short-term responses and long-term adaptations to cope with the frequent changes in the quality and quantity of incident light (Foyer et al., 2012).

One of the fast responses is NPQ, a process wherein the over-excitation in photosystem (PS) II is either dissipated as heat (Szabó et al., 2005; Lambrev et al., 2012; Ruban et al., 2012) or redistributed to PSI (Allen, 2003a). Another effective fast regulation to dissipate over-excitation is the transition of the linear electron transfer (LEF) to alternative electron transfer pathways, such as cyclic electron transport (CEF) so that ATP will be produced without NADPH generation, which alleviates the excitation pressure in the PSs and reduces the production of ROS (Heber et al., 1995; Heber, 2002; Johnson, 2005). In higher plants, CEF can consume up to 30% of photosynthetic electrons, and larger proportion of photosynthetic electrons were used in CEF in the green alga especially in the light stress conditions (Wagner et al., 2006; Wilhelm and Selmar, 2011). Turnover of the PSII supercomplexes can be regarded as one of the most important protective responses in the thylakoid membrane under strong light intensities (Kanervo et al., 2005). Under strong light conditions, PSII RC is the primary target and frequently undergoes degradation that leads to the reduction in PSII photochemical efficiency (Aro et al., 1993; Andersson and Barber, 1996; Andersson and Aro, 2001; Edelman and Mattoo, 2008). The photodamaged PSII RCs could be repaired through an efficient mechanism including degradation of the impaired D1 protein and reassembly of a *de novo* synthesized D1 protein into the thylakoid membrane, thus guarantee the maintenance of the PSII function (Aro et al., 1993, 2005; Nixon et al., 2005, 2010).

Natural selection under extreme light conditions resulted in long-term adaptation in different plant species showing specific characteristics, such as shade and sun plants, living on the forest floor and in open canopy, respectively. The shade-type chloroplasts are characterized by more and larger granal stacks, a higher stacking degree of thylakoids and less non-appressed membrane region than the sun-type chloroplasts (Meier and Lichtenthaler, 1981). In addition, shade or low-light grown plants regulate physiological photosynthetic unit, which results in different PS stoichiometry and antenna cross-section (Wilhelm and Wild, 1984; Anderson et al., 1988; Akoumianaki-Ioannidou et al., 2004). It was well-established that the photosynthesis of sun- or high-light plants is saturated at higher light levels, and exhibits greater high-light tolerance than that of shade- or low-light-acclimated plants (Öquist et al., 1992a,b; Dos Anjos et al., 2012; Lichtenthaler et al., 2013). Besides the responses to high irradiance at the photosynthetic level, the changes in the molecular level, both the genomic and proteomic changes in plants, are also involved in response to the high irradiance, not only in terms of short-term responses, but also in terms of long-term adaptations to environmental conditions (Rossel et al., 2002; Kimura et al., 2003; Phee et al., 2004; Murchie et al., 2005; Giacomelli et al., 2006).

In the present study, we provide deeper insights into the molecular mechanisms of the highly efficient photosynthesis on the primary reaction level in ephemerals adapting to high irradiances. By studying the behavior of three different C_3_ plant species, namely, *A. pumila*, *S. altissimum*, and *A. thaliana*, we concluded that the unusually stable PSII structure is one of the important adaptation mechanisms for the ephemeral plants sustaining extreme light condition.

## Materials and Methods

### Plant Materials and Growth Conditions

Seeds of the two spring ephemerals (*A. pumila* and *S. altissimum*) were collected in their original growing area in the southern margin of the Gurbantunggut Desert in the Dzungaria Basin in northern Xinjiang Uygur autonomous region, northwestern China, where the mean annual precipitation is less than 150 mm, occurring predominantly in the spring and early summer and yearly irradiance can reach around 2138 MJ m^−2^ during the growth season, and mean annual temperature is 7.3°C, sometimes up to 40.5°C. Seeds of *A. pumila*, *S. altissimum*, and *A. thaliana* were imbibed in the dark for 2 days at 4°C to ensure synchronized germination, and then sown and transferred to growth chambers (100 μmol photons m^−2^ s^−1^, and 12 h light/12 h dark at 22°C, 50–70% relative humidity) for the first 2 weeks. Then, the seedlings were transferred to and cultured in light of 100 or 600 μmol photons m^−2^ s^−1^ for the LL- or HL-treatment, respectively.

### Thylakoid Membrane Preparation

Thylakoid membranes used for gel analysis and western blot assay were prepared as described in Zhang et al. (1999). Briefly, leaves were homogenized in an ice-cold isolation buffer containing 400 mM sucrose, 50 mM HEPES-KOH (pH 7.8), 10 mM NaCl, and 2 mM MgCl_2_ and filtrated through two layers of cheesecloth. The filtrate was centrifuged at 5000 *g* for 10 min. The pellets were washed twice with isolation buffer and finally resuspended in the same buffer. The Chl content was determined spectrophotometrically according to Porra et al. (1989).

### SDS-PAGE and Immunoblot Analysis

Thylakoid membrane protein components and the dynamic changes under different light conditions were analyzed with 15% SDS polyacrylamide gels containing 6 M urea (Laemmli, 1970) and western blot assays with the standard protocol. After electrophoresis, the proteins were transferred onto nitrocellulose membranes and signified by probing with the specific primary antibodies. DyLight^TM^ 800 labeled secondary antibody (Kirkegaard & Perry Laboratories, Inc., USA) was used for infrared visualization of protein bands. Quantification of proteins was done with the Odyssey Infrared Imaging System (Li-COR Biosciences, Lincoln, NE, USA).

### Chlorophyll *a* Fluorescence Measurements

Chlorophyll *a* fluorescence parameters, including analysis of light intensity response curves, kinetics of NPQ induction and relaxation, as well as the PSII activity changes during strong light exposure, were measured using a PAM 2000 portable Chl fluorometer (Heinz-Walz, Germany) with a leaf-clip holder (2030-B) attached to leaves. The plants were dark-adapted for 30 min before measurements. Minimum fluorescence intensity (*F*_0_) was measured under a weak ML (wavelength 650 nm) at a light intensity of 0.5 μmol photons m^−2^ s^−1^. A saturating pulse (SP) of white light (4500 μmol photons m^−2^ s^−1^ for 0.8 s) was applied to the leaf to estimate the maximum fluorescence in the dark-adapted state (*F*_m_) and during illumination with AL (*F*_m_′). The steady-state fluorescence (*F*_t_) was recorded during AL illumination as well. The maximum quantum efficiency (*F*_v_/*F*_m_) was calculated from the ratio of variable (*F*_v_) to maximum fluorescence [*F*_v_/*F*_m_ = (*F*_m_−*F*_0_)/*F*_m_]. The quantum yield of PS II [Y(II)] was calculated as Y(II) = (*F*_m_′−*F*_t_)/*F*_m_′. The rETR was calculated according to the equation: rETR = Y(II)⋅PAR (PAR: photosynthetic active radiation, measured as μmol photons m^−2^ s^−1^). To eliminate the effects of the altered optical properties on the evaluation of rETR value in different samples, the light-saturation index *E*_k_, which is independent of leaf absorptivity, was used to evaluate the rETR, and it was determined from the intercept point of α- slope and *P*_max_: *E*_k_ = *P*_max_/α-slope, where *P*_max_ is the light-saturated rate of photosynthesis (the maximum of rETR in our calculation), and α-slope was calculated from the linear rise of the photosynthesis rate versus irradiance (Blache et al., 2011). NPQ was calculated as (*F*_m_−*F*_m_′)/*F*_m_′ (Maxwell and Johnson, 2000).

### Analysis of Fluorescence Induction Kinetics

Fast Chl *a* fluorescence induction kinetics were measured with a Plant Efficiency Analyser (PEA, Hansatech Instruments Ltd., King’s Lynn, Norfolk, UK) according to Strasser et al. (1995). Prior to the measurements, plants were dark-adapted for 30 min. The fluorescence was excited using a red light of 3500 μmol photons m^−2^ s^−1^ provided by an array of six light-emitting diodes (peak 650 nm) and focused on an area of 4 mm diameter, recorded during a time span from 10 μs to 1 s. The fluorescence signal at 50 μs was taken as *F*_0_ (the O point) and the point that the fluorescence reach the maximum (*F*_m_) was defined as the P point. A number of phases were visible on a log_10_ time scale to rise to *F*_m_: a first rise from the origin (O) to an intermediate step (J step, at 2 ms) and then a second slower rise involving a second intermediate (I step, at 30 ms) to a peak (P; Strauss et al., 2006). To analyze the fluorescence induction curve in detail, we calculated the integrated area between the measured fluorescence signal (*F*_t_) and the *F*_m_ as the following equation:

$$\mathrm{area}=\int_{0}^{Tm}\left(F_{m}-F_{t}\right)\mathrm{dt}$$

*T*_m_ is the illumination time needed to reach *F*_m_. The parameter *S*_m_, a measure of the energy needed to close the active PSII RC, was calculated by dividing the area by (*F*_m_−*F*_0_): *S*_m_ = area/(*F*_m_−*F*_0_; Strasser et al., 2000).

### Measurements of P_700_ Redox Kinetics

The redox changes of P_700_ were assessed by monitoring absorbance at 820 nm, using a PAM 101 fluorometer (Heinz-Walz, Germany) equipped with an emitter-detector unit (ED 800T). The measurement was performed as previously described (Meurer et al., 1996). The measurement of the relative amount of total photooxidizable P_700_ was completed by superimposing a saturating white light pulse (3000 μmol photons m^−2^ s^−1^ for 0.8 s) onto the far red (FR) light background. Dark re-reduction kinetics of P_700_^+^ were measured after a period of FR light in the presence of 50 μM 3-(3,4-dichlorophenyl)-1,1-dimethylurea (DCMU) to inhibit the electrons from PSII.

### Quantum Yield of PSII and PSI

In order to reveal the relationship between the functional PSII and PSI in the different plant species grown under different light conditions, a Dual-PAM-100 measuring system (Heinz Walz GmbH, Effeltrich, Germany) was employed to assess the Y(II) and Y(I) according to the previously published method (Klughammer and Schreiber, 2008; Pfündel et al., 2008). All samples were dark-adapted for 30 min before measurements. The minimal fluorescence (*F*_0_) was measured in the dark-adaptation state with a weak ML. An SP was then applied to detect the maximum fluorescence (*F*_m_). Subsequently, FR light was switched on for 10 s to determine the maximal P_700_ change (*P*_m_). Then, after the delay time of 40 s, the AL at 90 μmol photons m^−2^ s^−1^ was switched on. After the SP2 delay time of 1 s, another SP was applied, followed by further SP applied every 20 s after the onset of the AL to determinate the maximum fluorescence signal (*F*_m_′) and the maximum *P*_700_^+^ signal (*P*_m_′) under AL without FR illumination. The P_700_^+^ signal (P) was recorded just before an SP, which was applied to determine *F*_m_′ and *P*_m_′. The slow induction curves were recorded for 400 s to achieve the steady state, and afterwards the AL was turned off.

Y(II) and Y(I) were calculated according to Suzuki et al. (2011), and the quantum yield of cyclic electron transfer around PSI [Y(CEF)] according to Huang et al. (2010), which are presented in the following equations:

$$\begin{array}{c} Y\left(\mathrm{II}\right)=\left(F_{m^{\prime }}-F\right)/F_{m^{\prime }},\\ Y\left(I\right)=\left(P_{m^{\prime }}-P\right)/P_{m},\\ Y\left(\mathrm{CEF}\right)=Y\left(I\right)-Y\left(\mathrm{II}\right). \end{array}$$

### Photoinhibition Analysis

Detached leaves of the LL-grown plants, kept floating adaxial side up on water, were illuminated at a photon density of 2000 μmol photons m^−2^ s^−1^, and Chl *a* fluorescence was measured using a PAM 2000 portable Chl fluorometer (Heinz-Walz, Germany). To examine the effect of inhibition of the chloroplast-encoded protein synthesis, detached leaves were treated with 1 mM lincomycin at an irradiance of 20 μmol photons m^−2^ s^−1^ for 3 h to block protein synthesis and then transferred to the irradiance of 2000 μmol photons m^−2^ s^−1^ to carry out photoinhibitory treatment with lincomycin existing during the whole process. The temperature was maintained at 22°C during the photoinhibition treatment.

### Oxygen Evolution Measurement

Thylakoid membranes used for oxygen evolution measurements were isolated as described by Allahverdiyeva et al. (2007). Light-saturated oxygen evolution rate from thylakoids was measured with a Clark-type oxygen electrode (Hansatech, King’s Lynn, UK) at 25°C and in the presence of 0.1 mM 2,6-dichloro-p-benzoquinone (DCBQ) as an artificial electron acceptor.

## Results

### Plant Materials

**FIGURE 1:**
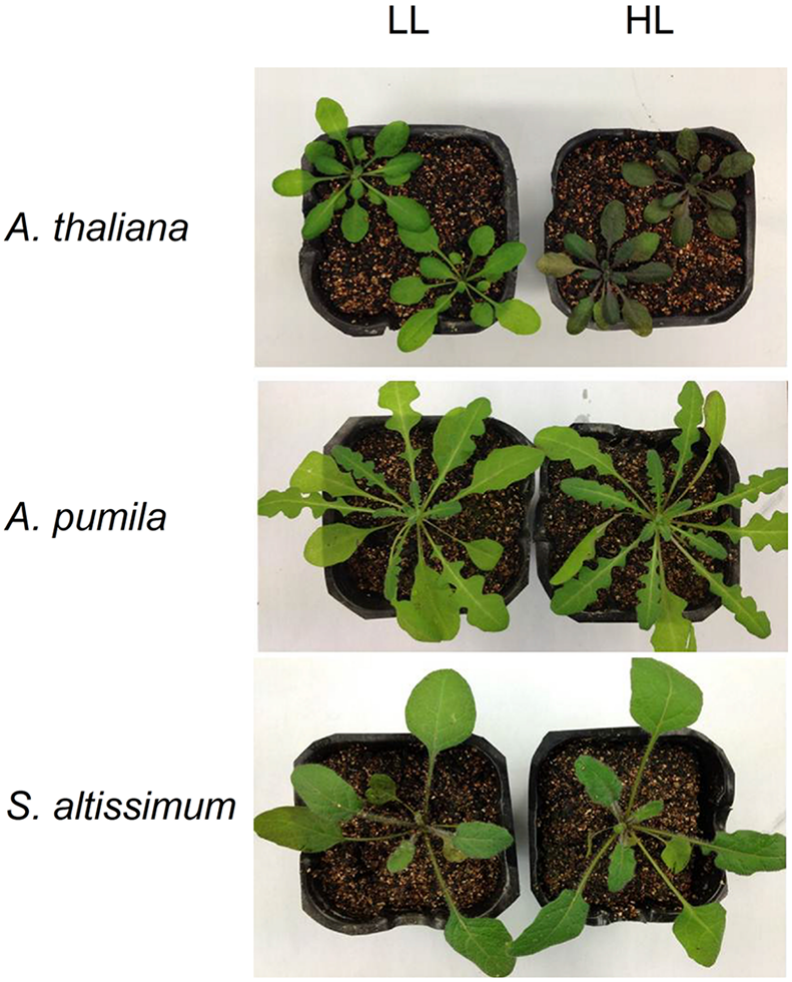
**Phenotype of *Arabidopsis thaliana*, *A. pumila* and *Sisymbrium altissimum* grown under different light conditions**. All plants were grown in 12 h light/12 h dark photoperiod. LL- and HL-plants were grown in low light (LL, 100 μmol photons m^−2^ s^−1^ for 4 weeks) and high light (HL, 100 μmol photons m^−2^ s^−1^ for 2 weeks and 600 μmol photons m^−2^ s^−1^ for the following 2 weeks) conditions, respectively.

**FIGURE 2:**
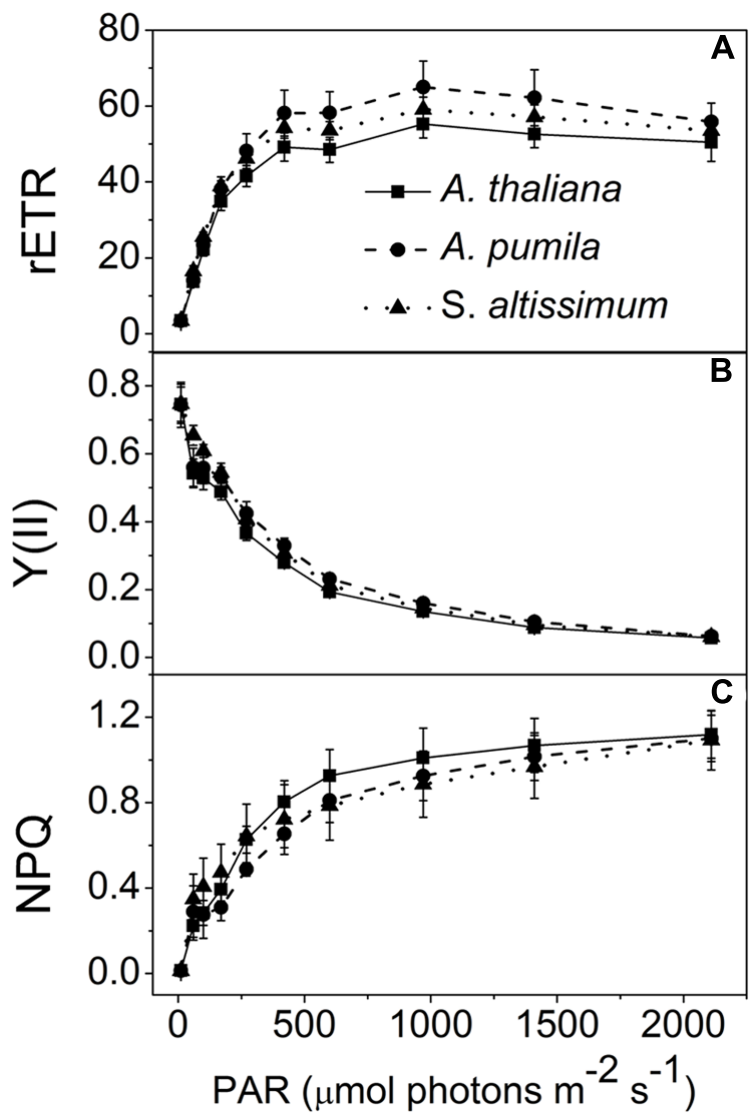
**Analysis of PSII functions in ephemerals and *A. thaliana* grown under LL conditions. (A–C)**, Light intensity dependence of the relative electron transport rate through PSII (rETR; **A**); the quantum yield of electron transport at PSII [Y(II)] **(B)**; and non-photochemical quenching (NPQ; **C**). Data are means ± SD from five independent measurements.

In this study, the photosynthetic characteristics of three species of the Cruciferae family grown under different light conditions (LL and HL) were investigated. All the plants grew well at an irradiance of 100 μmol photons m^−2^ s^−1^ (**Figure 1**), with a typical *F*_v_/*F*_m_ value for higher plants (ca. 0.83, **Table 1**). The PSII function of all the three species in LL showed indistinguishable differences responding to the increasing light intensities (**Figure 2**). rETR of LL-grown plants showed similar light dependent kinetics, and reached the maximum at around 600 μmol photons m^−2^ s^−1^ (**Figure 2A**). The light intensity dependent changes in Y(II) and NPQ in all LL-grown plants presented similar trends and extents (**Figures 2B,C**).

**Table 1:** The maximal photochemical efficiency of PSII (*F*_v_/*F*_m_) and the characteristic light intensity (*E*_k_; μmol photons m^−2^ s^−1^) calculated from rETR in *Arabidopsis thaliana* and ephemerals grown under different light conditions.

	* F*_v_/*F*_m_	*E*_k_
*A. thaliana* LL	0.83 ± 0.01	174
*A. thaliana* HL	0.69 ± 0.05∗	140
*A. pumila* LL	0.81 ± 0.01	202
*A. pumila* HL	0.82 ± 0.01	290
*S. altissimum* LL	0.82 ± 0.01	166
*S. altissimum* HL	0.83 ± 0.01	238

*Data in the table are means ± SD from five independent experiments. The significant difference according to Student’s *t*−test (*P* < 0.05) is marked with an asterisk in the same column*.

### High Light Conditions Enhanced the Photosynthetic Electron Transport in the Ephemerals

**FIGURE 3:**
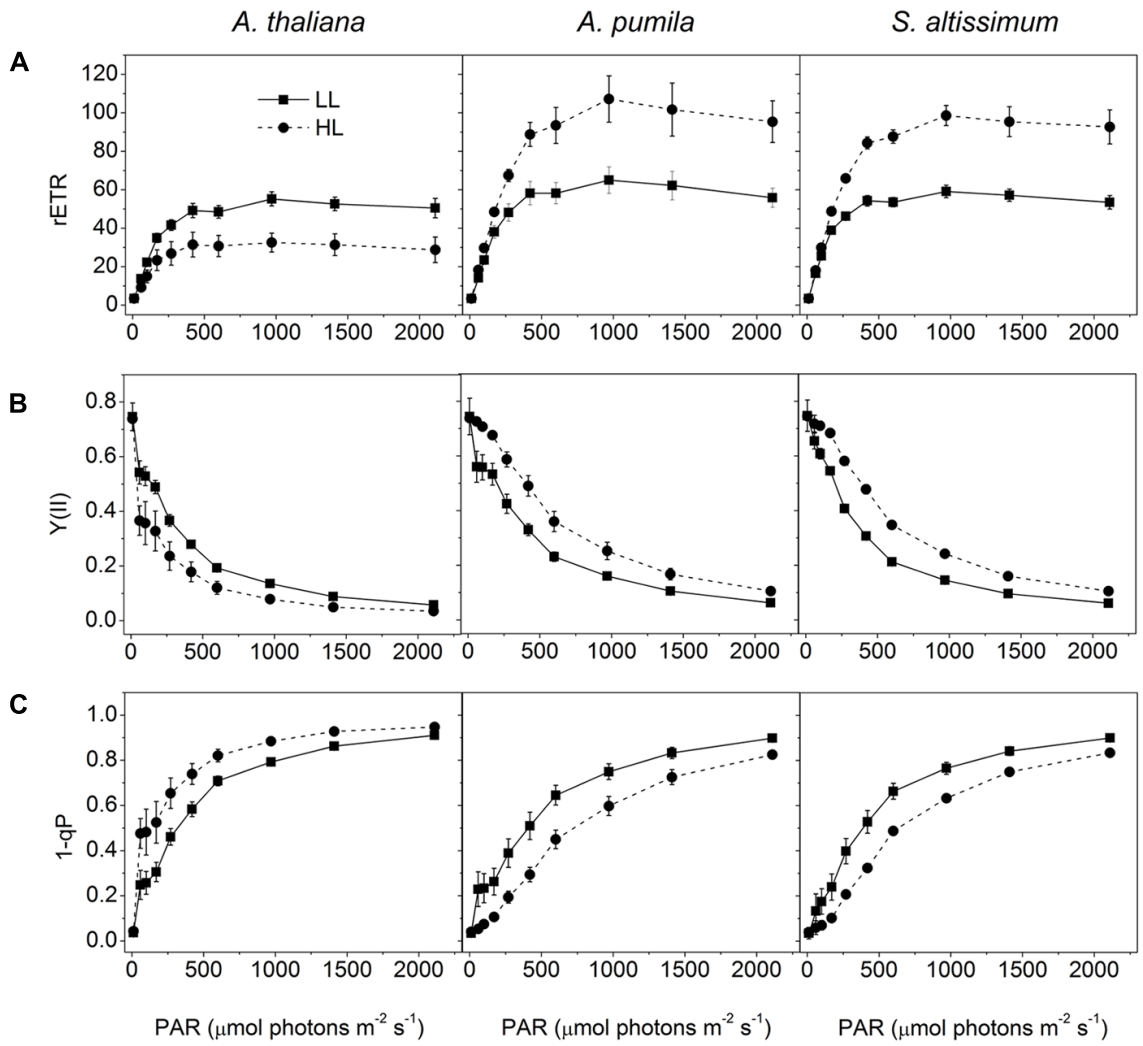
**Analysis of PSII functions in ephemerals and *A. thaliana* grown under different light conditions. (A–C)**, Light intensity dependence of rETR **(A)**; Y(II) **(B)**; and the closed reaction centers of PSII (1-qP) **(C)**; of ephemerals and *A. thaliana* grown in LL and HL intensities. Data are means ± SD from five independent measurements.

When grown under HL conditions, *A. pumila* and *S. altissimum* showed no difference in phenotype compared to the LL-grown ones, while *A. thaliana* already exhibited symptoms of photoinhibition (**Figure 1**), accompanied by decreased Chl contents and Chl *a/b* ratio (**Table 2**) implying reduced PSII RCs, the significant drop in the *F*_v_/*F*_m_ value (0.69, **Table 1**) and concomitant reduction in PSII efficiency. HL-grown *A. thaliana* experienced a higher excitation pressure with more closed PSII RCs, as indicated by the higher 1-qP value, a measure of the fraction of Q_A_ reduced (**Figure 3C**). Consistent with this, the Y(II) were much lower and the rETR declined to almost half of those in the LL-grown ones (**Figures 3A,B**). In order to get rid of the possible effect resulting from the changes in the Chl content per area, as Chl content in the HL-grown *A. thaliana* was reduced to 16.12 μg Chl/cm^2^ from 19.49 μg Chl/cm^2^ leaf area in the LL-grown ones, while those in the HL-grown ephemerals increased (**Table 2**), the *E*_k_ values of different samples, which indicate the onset of light saturation, were calculated according to Blache et al. (2011). Comparing the plants grown in LL and HL conditions, the *E*_k_ values were reduced from 174 to 140 in *A. thaliana*, increased from 202 to 290, and from 166 to 238 in *A. pumila* and *S. altissimum*, respectively (**Table 1**). Therefore, it is reasonable to propose that the reduced rETR in the HL-grown *A. thaliana* are the consequence of the altered integrity, or photoinhibition, of the PSII supercomplexes. Unlike *A. thaliana*, which presented decreased PSII activities, *A. pumila* and *S. altissimum* presented even higher rETR, *E*_k_ and Y(II), and much lower excitation pressure, which is an indicator of a high redox state of Q_A_, with less closed PSII RCs under HL conditions (**Figure 3**; **Table 1**).

**Table 2:** Chlorophyll (Chl) contents of dark-adapted leaf tissue in *A. thaliana* and ephemerals grown under different light conditions.

	μg Chl/cm^2^	Chl *a/b*
*A. thaliana* LL	19.79 ± 1.29	3.11 ± 0.02
*A. thaliana* HL	16.12 ± 0.37	3.00 ± 0.02
*A. pumila* LL	17.41 ± 2.35	3.10 ± 0.03
*A. pumila* HL	18.27 ± 3.54	3.02 ± 0.03
*S. altissimum* LL	20.64 ± 2.76	3.39 ± 0.09
*S.altissimum* HL	22.85 ± 1.11	3.43 ± 0.04

*Data in the table are means ± SD from three independent experiments*.

**FIGURE 4:**
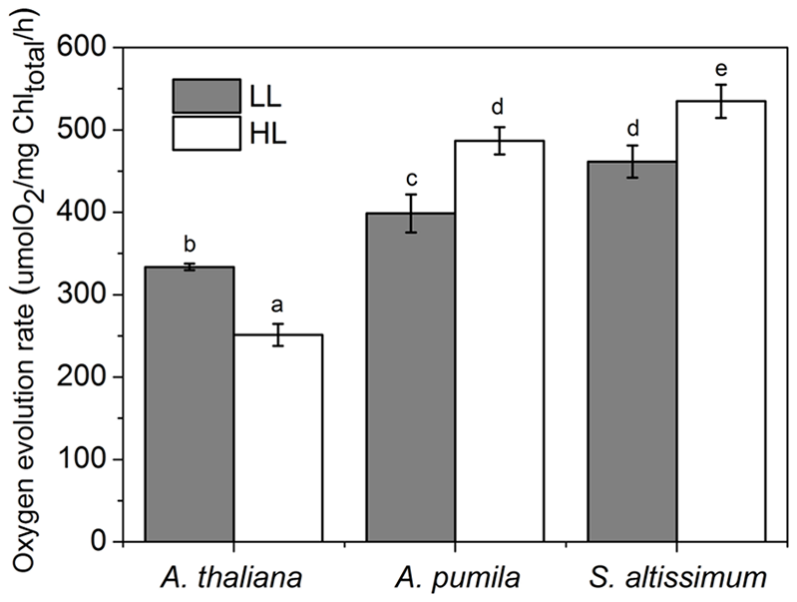
**Oxygen evolution activities of thylakoid membranes in ephemerals and *A. thaliana* grown under different light conditions**. Oxygen evolution activities were measured in isolated thylakoids using DCBQ as the artificial electron acceptor from PSII. All values are means ± SD from five independent measurements. Significant differences according to Student’s *t*−test (*P* < 0.05) were marked with different letters.

Oxygen evolution activity was measured in isolated thylakoids using DCBQ as the artificial electron acceptor for PSII. The oxygen evolution rate in LL-grown *A. thaliana* was 333 ± 4.86 μmol O_2_/mg Chl/h, which was consistent with previous results (Suorsa et al., 2006), and decreased remarkably (dropped to 70% of the LL-grown ones) under HL conditions, while that in HL-grown *A. pumila* and *S. altissimum* increased obviously compared to that in the LL-grown ones (**Figure 4**).

In conclusion, PSII of the ephemerals *A. pumila* and *S. altissimum* showed even higher photochemical activities under HL conditions, when *A. thaliana* already suffered from obvious photoinhibition.

### High Light Conditions did not Inhibit the Electron Transport Downstream of Q_A_ in Ephemerals

**FIGURE 5:**
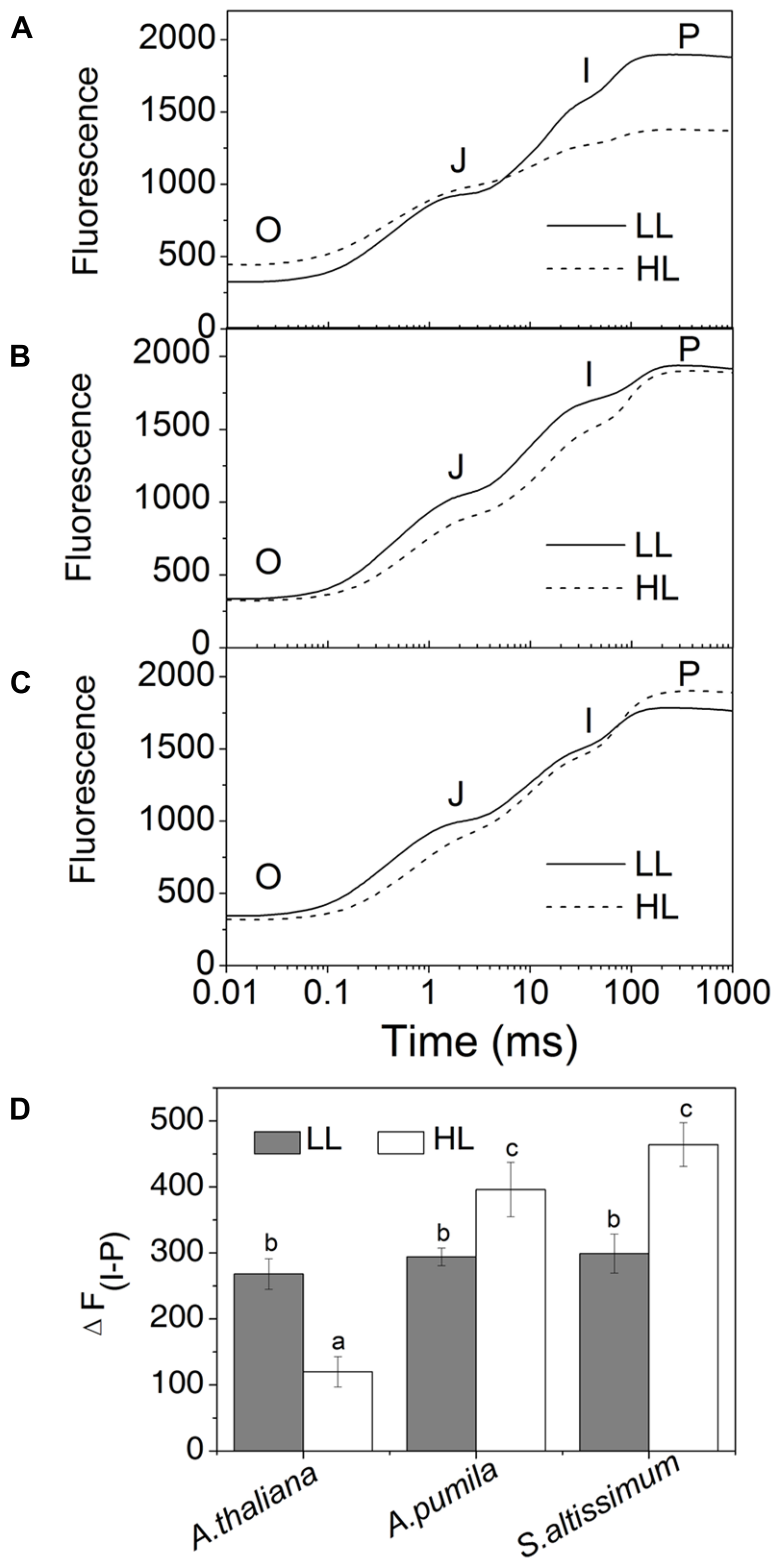
**The fast fluorescence induction kinetics of ephemerals and *A. thaliana* grown at different light intensities. (A–C)**, Fluorescence rise in *A. thaliana* **(A)** and the ephemerals *A. pumila* **(B)**, *S. altissimum* **(C)** grown at LL and HL intensities, which was induced on dark-adapted leaves using a saturating flash of white light (3500 μmol photons m^−2^ s^−1^). The measurements were repeated for eight times. **(D)** Fluorescence changes of the I-P phase in the rapid fluorescence induction kinetics of ephemerals and *A. thaliana* grown in different light conditions. Data are means ± SD from eight independent measurements. Significant differences according to Student’s *t*−test (*P* < 0.05) were marked with different letters.

In order to obtain deeper insights into the light harvesting and electron transport activities of the two ephemerals, the fast Chl *a* fluorescence transient of the dark-adapted plants grown under different light intensities was analyzed. Under LL conditions, the fast Chl *a* fluorescence transient of all the three species showed similar tendency. However, different characteristics were presented in the HL-grown plants (**Figures 5A–C**). It is obvious that the HL-grown *A. thaliana* showed a higher *F*_0_ value (**Table 3**), which implied a less effective usage of the energy absorbed by antenna system for charge separation in the PSII RCs of the HL-grown *A. thaliana*. Further analysis revealed that the O–J and J–P phase increased faster in the HL-grown ephemerals than those in the LL-grown ones, while *A. thaliana* showed a completely reversed tendency (**Figures 5A–C**). There is a correlation between the Chl *a* fluorescence transients and the reduction of the PQ pool, and the O–J phase is the photochemical phase, representing the reduction of Q_A_ to Q_A_^−^ (Strasser et al., 1995). Then Q_B_, the next electron acceptor is reduced by Q_A_^−^ twice. The intermediate step I is suggested to be related to a heterogeneity in the redox states of Q_B_, which is due to the existence of fast and slow reducing PQ centers (Strasser et al., 1995; Hill et al., 2004). The P peak is ascribed to the filling of the PQ pool and reaches when all the PQ molecules are reduced to PQH_2_ (Strasser et al., 1995; Hill et al., 2004). The Chl *a* fluorescence transients revealed clearly that the electron transport downstream of Q_A_ accelerated in HL-grown ephemerals, while the ability of electron transport downstream of Q_A_ was impaired in HL-grown *A. thaliana*. **Table 3** showed that *A. thaliana* had lower *S*_m_/*T*_m_ value, indicating more closed RCs in HL, while RCs of the two ephemerals were kept open under HL conditions. This result was in agreement with the higher PSII activities, a phenomenon related to the more oxidized PQ, as indicated by the higher *S*_m_ value, a parameter representing a measurement of energy needed to close the active PSII RC, in the two ephemerals grown under HL conditions (**Table 3**).

**Table 3:** Analysis of Chl fluorescence parameters in *A. thaliana* and ephemerals grown under different light conditions.

	*F*_0_	*S*_m_	*S*_m_/*T*_m_
*A. thaliana* LL	325 ± 18^a^	19.58 ± 0.95^b^	0.072 ± 0.001^b^
*A. thaliana* HL	445 ± 64^b^	12.16 ± 0.80^a^	0.048 ± 0.005^a^
*A. pumila* LL	338 ± 5^a^	20.22 ± 0.61^b^	0.072 ± 0.005^b^
*A. pumila* HL	326 ± 14^a^	24.81 ± 0.79^c^	0.083 ± 0.012^b^
*S. altissimum* LL	347 ± 31^a^	26.57 ± 1.15^c^	0.072 ± 0.007^b^
*S. altissimum* HL	321 ± 8^a^	28.96 ± 0.56^d^	0.072 ± 0.012^b^

*Values in the table are means ± SD from eight independent experiments. Values marked with different letters in the same column were significantly different according to Student’s *t*−test (*P* < 0.05)*.

### NPQ in Ephemerals Increased Only Slightly under High Light Conditions

**FIGURE 6:**
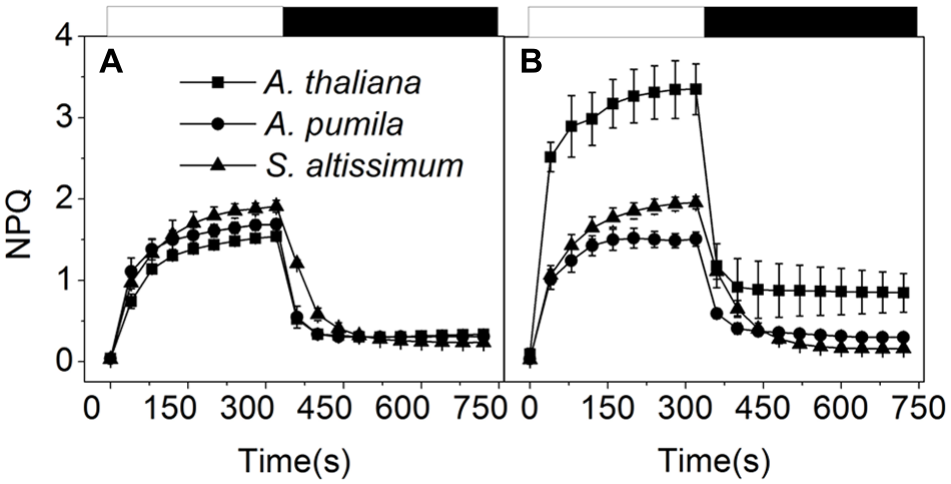
**Non-photochemical quenching analysis of ephemerals and *A. thaliana* grown at different light intensities**. Kinetics of NPQ induction and relaxation were recorded with a pulse amplitude modulated fluorometer. Chl fluorescence was measured in intact, dark-adapted leaves during 6 min of illumination at 1200 μmol photons m^−2^ s^−1^ followed by 6 min of dark relaxation. **(A,B)**, Kinetics of NPQ induction and relaxation in LL-grown **(A)** and HL-grown **(B)** ephemerals and *A. thaliana*. Data represented are means ± SD of five independent measurements.

To find out how ephemerals keep increased electron transport flow under HL conditions, the NPQ kinetics in LL- or HL-grown plants were recorded (**Figure 6**). When grown under LL conditions, the three species showed similar NPQ kinetics (**Figure 6A**), while significantly different NPQ kinetics were observed under HL conditions. NPQ levels in the HL-grown *A. pumila* and *S. altissimum* remained almost unchanged while that of *A. thaliana* was almost double, compared with those in the LL-grown ones. Upon dark relaxation, HL-grown *A. thaliana* still held a higher NPQ level than the two ephemerals (**Figure 6B**), which indicated clearly that the relative slower NPQ processes (state transition quenching, qT, or photoinhibitory quenching, qI) were triggered in *A. thaliana* under HL conditions.

### The Ephemerals Showed Lower Photoinhibition Rate under Photoinhibitory Irradiance

Photoinhibition of PSII appears when the repair of PSII is not efficient enough to keep up with the rate of damage. Detached leaves of the three species grown in LL were exposed to photoinhibitory irradiance at a light intensity of 2000 μmol photons m^−2^ s^−1^, in the presence or absence of lincomycin, an antibiotic that blocks the synthesis of chloroplast-encoded proteins, and changes of PSII photochemical activities, in view of the relative changes of photochemical efficiency of PSII, and PSII structures, in view of the changes of different protein subunit contents, were monitored.

**FIGURE 7:**
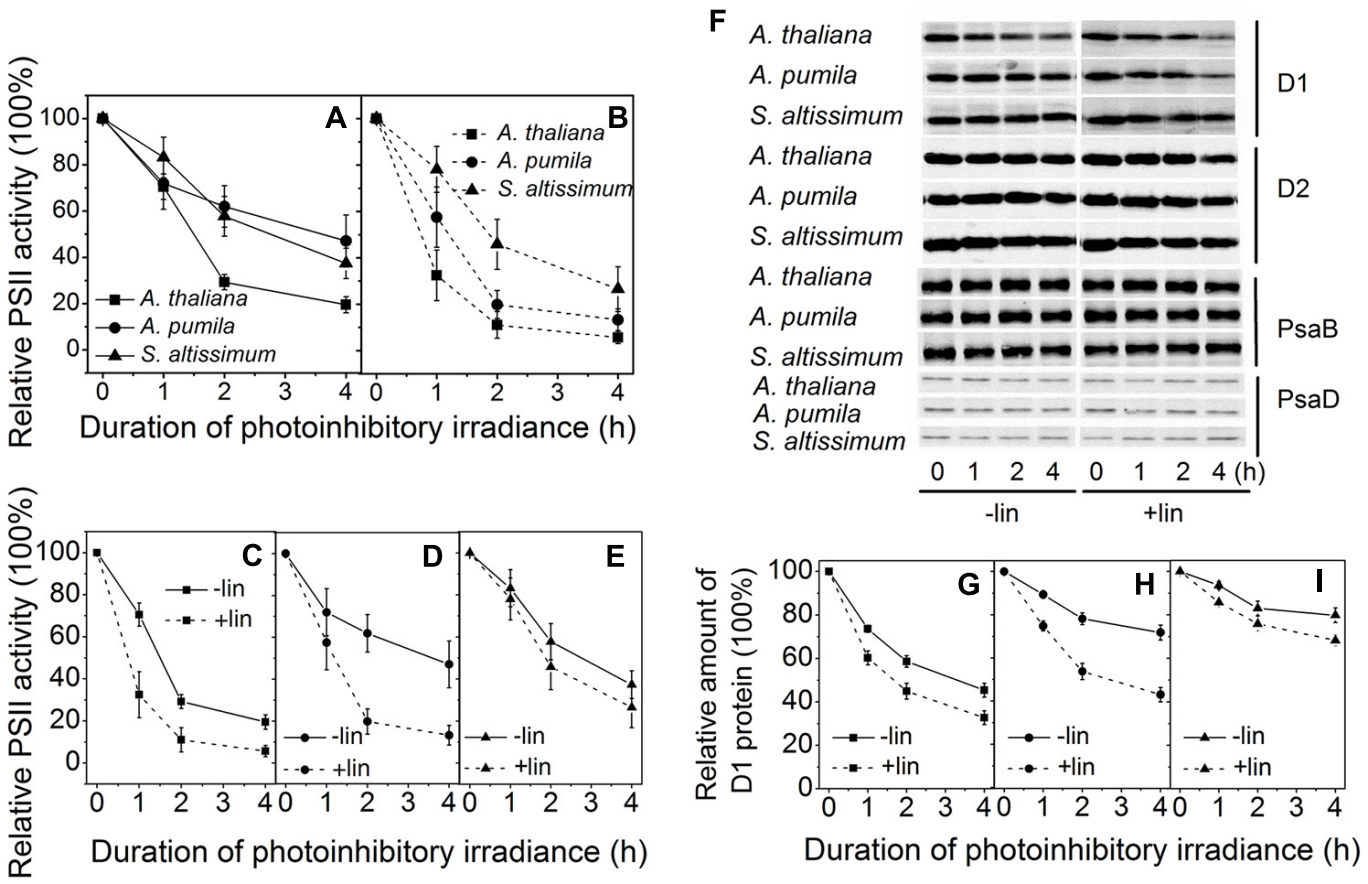
**Changes of PSII activities and thylakoid protein contents in LL-grown ephemerals and *A. thaliana* during strong light illumination. (A,B)**, The maximal photochemical efficiency of PSII (*F*_v_/*F*_m_) was measured for detached leaves from ephemerals and *A. thaliana* during exposure to irradiance of 2000 μmol photons m^−2^ s^−1^ in the absence **(A)** or presence **(B)** of lincomycin. **(C–E)**, Changes of *F*_v_/*F*_m_ in detached leaves in *A. thaliana* **(C)**, *A. pumila* **(D)** and *S. altissimum* **(E)** during exposure to irradiance of 2000 μmol photons m^−2^ s^−1^ in the absence and presence of lincomycin. **(F)** Changes of thylakoid protein contents in ephemerals and *A. thaliana* during strong light illumination. Thylakoid protein samples were extracted from detached leaves having been subjected to 2000 μmol photons m^−2^ s^−1^ irradiance for different time in the absence and presence of lincomycin, and then separated by SDS-PAGE and immunodetected with specific antibodies against D1, D2, PsaB, and PsaD. Equivalent thylakoid membrane proteins (with 1 μg Chl) were loaded. **(G–I)** Quantification of D1 proteins by immunoblotting in *A. thaliana* **(G)**, *A. pumila* **(H)**, and *S. altissimum* **(I)** during exposure to irradiance of 2000 μmol photons m^−2^ s^−1^ in the absence and presence of lincomycin. The amount of the D1 protein before treatment was defined as 100%.

**Figures 7A–E** shows the time course of the *F*_v_/*F*_m_ decline upon the photoinhibitory irradiance treatment. It is clear to see that after 2 h illumination, the *F*_v_/*F*_m_ in *A. thaliana*, *A. pumila*, and *S. altissimum* declined to about 30%, 60 and 60% respectively, and declined further to 20, 50, and 40% of the initial values, respectively, after 4 h illumination in the absence of lincomycin. These results demonstrated that PSII complexes in both ephemerals are less sensitive to the photoinhibitory strong light (**Figure 7A**). In the presence of lincomycin, the decline of *F*_v_/*F*_m_ in *A. thaliana* (to 10% of the original value after 4 h treatment) was faster than those observed in *A. pumila* and *S. altissimum* (to 15 and 35% of the original values, respectively, after 4 h treatment; **Figure 7B**). From **Figures 7A,B**, it is clear to see that the ephemerals exhibited much less decline in *F*_v_/*F*_m_ value upon photoinhibitory irradiance treatment compared with *A. thaliana.* Comparing the time course of the *F*_v_/*F*_m_ decline in different species with or without lincomycin treatment reveals that *A. thaliana* showed remarkable PSII degradation and repair cycle upon onset of the photoinhibitory irradiance treatment (**Figure 7C**), as the leaves showed significantly different *F*_v_/*F*_m_ values at the first hour of the treatment. Both the ephemerals showed less reduction in *F*_v_/*F*_m_ values during strong light treatment, however, they followed different mechanisms to sustain high PSII photochemical activities. *A*. *pumila* sustained high PSII activity via very efficient D1 protein synthesis, while *S*. *altissimum* via its resistance to D1 protein photodegradation (**Figures 7D,E**). The conclusion drawn from *F*_v_/*F*_m_ decline processes in the three species could be confirmed by the dynamic changes of D1 protein after exposure to strong light illumination in the presence and absence of lincomycin (**Figures 7F–I**). As shown in **Figures 7F,G**, the content of D1 protein decreased gradually in *A. thaliana* during all time course of strong light treatment, and lincomycin treatment accelerated the decline in D1 protein content. The content of D1 protein in *A. pumila* decreased slightly in the absence of lincomycin, but to similar degree as in *A. thaliana* in the presence of lincomycin (**Figures 7F,H**). *S*. *altissimum* showed only slight D1 protein decline after 4 h exposure to the strong light both in the presence and absence of lincomycin (**Figures 7F,I**). The variation of D1 protein degradation in different species, in the presence of lincomycin, highlighted clearly that photodegradation of D1 protein in *S. altissimum* progressed much slower than those in *A. pumila* and *A. thaliana*, and that the photodegradation of D1 protein was slightly slower in *A. pumila* than that in *A. thaliana*. The contents of D2 protein in the ephemerals and *A. thaliana* declined slightly during strong light exposure, especially in the presence of lincomycin, but obviously to a less extent compared with the D1 protein degradation. The levels of PSI RC proteins PsaB and PsaD were relatively stable in all the species during high irradiance treatment, no matter in the presence or absence of lincomycin (**Figure 7F**).

### The PSI Activities in Ephemerals Increased Enormously under High Light Conditions

**FIGURE 8:**
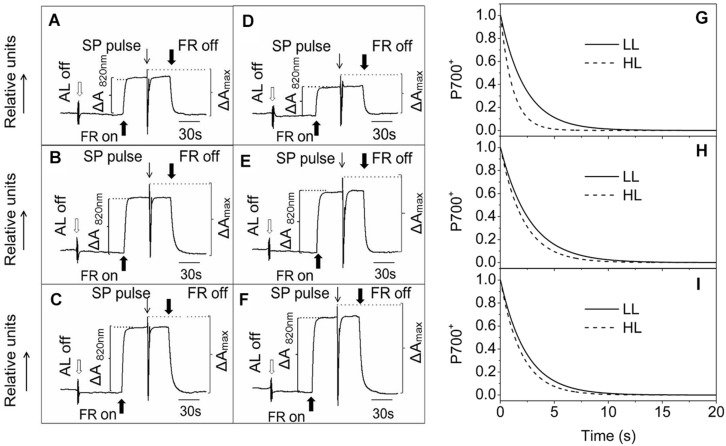
**Redox kinetics of P_700_ induced by far-red (FR) light in leaves of ephemerals and *A. thaliana* grown at LL and HL intensities**. The redox kinetics of P_700_ were investigated by measuring absorbance changes of P_700_ at 820 nm induced by FR light. **(A–C)**, the redox kinetics of P_700_ in leaves of *A. thaliana* **(A)**, *A. pumila* **(B)**, and *S. altissimum* **(C)** grown in LL conditions. **(D–F)**, the redox kinetics of P_700_ in leaves of *A. thaliana* **(D)**, *A. pumila* **(E)**, and *S. altissimum* **(F)** grown in HL conditions. FR, far-red light (720 nm, 15 μmol photons m^−2^ s^−1^). SP, saturating pulse (3000 μmol photons m^−2^ s^−1^). Δ*A*_820__nm_, relative amount of photooxidized P_700_ under FR illumination. Δ*A*_max_, relative amount of total photooxidizable P_700_. **(G–I)**, Dark re-reduction kinetics of P_700_^+^ after a FR light period measured on leaf disks of LL- and HL-grown *A. thaliana* **(G)**, *A. pumila* **(H)**, and *S. altissimum* **(I)** vacuum-infiltrated with 50 mM 3-(3,4-dichlorophenyl)-1,1-dimethylurea (DCMU). The measurements were repeated for three times and the representative curves were shown.

**FIGURE 9:**
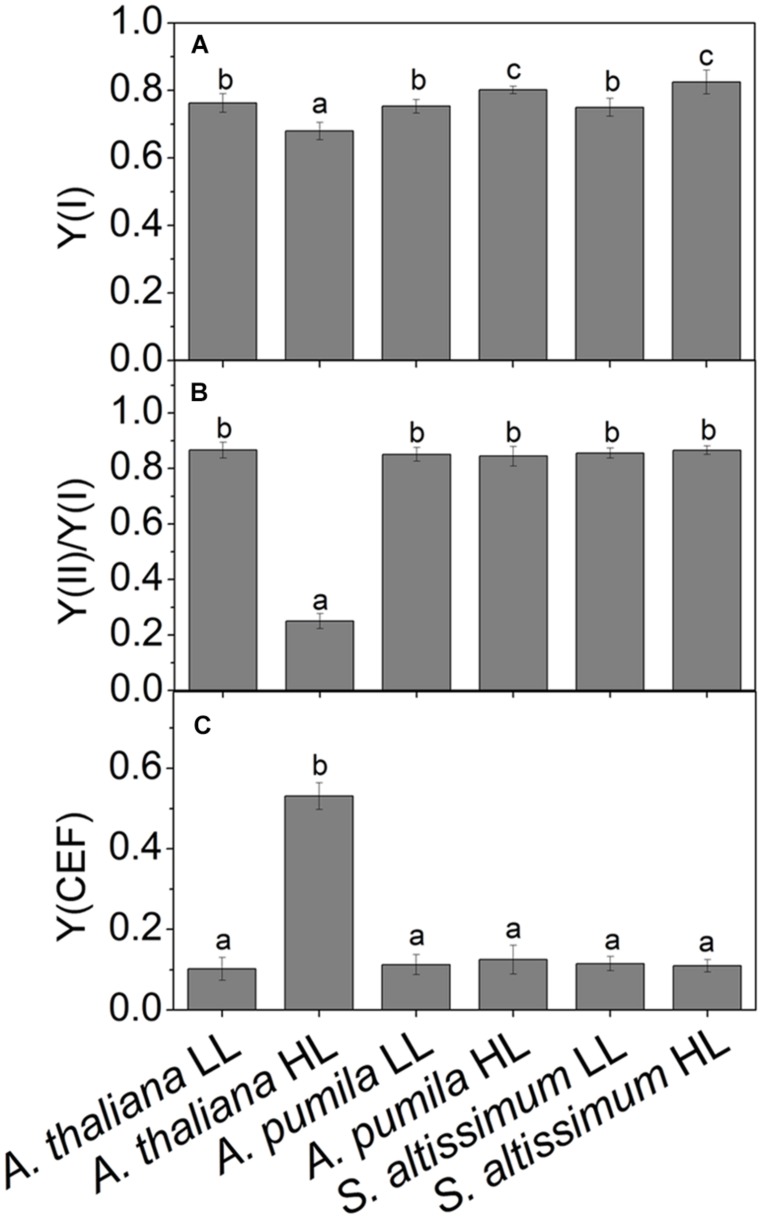
**Quantum yields of the two photosystems and CEF in ephemerals and *A. thaliana* grown at LL and HL intensities. (A)** The photochemical quantum yield of PSI [Y(I)]; **(B)** The ratios of Y(II)/Y(I); **(C)** The quantum yield of CEF [Y(CEF)] in LL- and HL-grown ephemerals and *A. thaliana*. All values are means ± SD from three independent measurements. Significant differences according to Student’s *t*−test (*P* < 0.05) were marked with different letters.

Photosynthetic control of electron transport routes in thylakoid is a fundamental process regulating the photochemical efficiency and carbon fixation. Previous studies showed that the I-P rise in the OJIP transient was closely correlated with PSI content and activity (Schansker et al., 2003, 2005). As we can see from **Figure 5**, the I-P rise of fluorescence transient in the ephemerals was higher in HL, while that of *A. thaliana* changed in the opposite trends, indicating that PSI activities in *A. thaliana* and the ephemerals should be different when grown in HL conditions. To check this possibility, we measured the FR light induced changes in the absorption at 820 nm using a PAM 101 fluorometer to monitor the redox state of P_700_ (Meurer et al., 1996). As shown in **Figures 8A–F**, the absorbance changes of P_700_ at 820 nm (ΔA_820_) of *A. pumila* and *S. altissimum* were higher than that of *A. thaliana* under both light conditions, indicating that the oxidization of P_700_ is generally higher in ephemerals. Calculating the difference in the absorption between the maximal P_700_ oxidization after a saturating light pulse and the lowest P_700_ oxidization after dark relaxation, we got the Δ*A*_max_ value that represents the total oxidizable P_700_, which is used to illustrate the maximum photochemical capacity of P_700_. As shown in **Figures 8A–F**, *A. thaliana* possessed relatively lower maximum P_700_ photochemical capacity under HL conditions, as indicated by the lower Δ*A*_max_ value compared to the LL-grown ones, while the two ephemerals possessed higher Δ*A*_max_ values, which indicated that more quanta were needed in the ephemerals, especially in the HL-grown ones. Quantum yield of energy conversion in PSI were also recorded by a Dual-PAM-100 measuring system to study the PSI activity (Detailed description of the simultaneous measurements of fluorescence and P_700_ signals was in Supplementary Figure S1). As seen in **Figure 9A**, Y(I) in the two HL-grown ephemerals was enhanced obviously compared with the LL-grown ones, while the value of Y(I) in HL-grown *A. thaliana* decreased to 90% of the LL-grown ones.

Analyzing the I-P rise in the OJIP transient (ΔF_I-P_), and the Δ*A*_820_ values, measured for estimating PSI activities under different light conditions, we can easily see that HL-grown *A. thaliana* and ephemerals behaved differently. Both ΔF_I-P_ and ΔA_820_ values in the ephemerals showed obvious increase under HL conditions compared with the LL-grown ones, while those of HL-grown *A. thaliana* decreased markedly (**Figures 5** and **8A–F**). This can be further corroborated by the direct estimation of the photochemical activities of PSII and PSI measured with a Dual-PAM measuring system. In accordance with the results of ΔF_I-P_ and ΔA_820_ values, both Y(II) and Y(I) increased markedly, and the ratio of Y(II)/Y(I) remained unchanged in the two ephemerals under both conditions, which verified that the relationship between PSII and PSI activities in the ephemerals were kept fairly stable even under HL conditions. On the other hand, the value of Y(II)/Y(I) in HL-grown *A. thaliana* decreased to only 30% of those in the LL-grown ones (**Figure 9B**), which implied severely impaired PSII activities and a significantly altered relationship between the functions of PSII and PSI in the HL-grown *A. thaliana*.

Besides LEF, other alternative electron transport pathways including pseudocyclic electron transfer or CEF are also involved in the electron flow via PSI (Allen, 2003b). Y(CEF) was similar in LL- and HL-grown ephemerals, while that in HL-grown *A. thaliana* enhanced more than four times (**Figure 9C**), indicating the significant induction of CEF in *A. thaliana* under HL conditions. CEF can be estimated indirectly by measuring the re-reduction rate of the P_700_^+^ after turning off the FR light in the presence of DCMU which inhibited electron donation from PSII (Joët et al., 2002). As **Figure 8G** shows, the re-reduction kinetics of P_700_^+^ in the dark after a FR period accelerated greatly in the HL-grown *A. thaliana* compared with the LL-grown ones, and the half-times (*t*_1/2_) of the dark re-reduction of P_700_^+^ were about 0.90 ± 0.09 s and 1.60 ± 0.14 s in HL- and LL-grown *A. thaliana*, respectively (**Table 4**). The P_700_^+^ re-reduction kinetics in the dark also accelerated slightly in the HL-grown ephemerals compared to that in the LL-grown ones, but to a far lesser extent than the HL-grown *A. thaliana* (**Figures 8H,I**; **Table 4**). The faster P_700_^+^ re-reduction rate in the presence of DCMU in the HL-grown *A. thaliana* also illustrated that CEF was significantly induced under over-excitation conditions.

**Table 4:** The half-times (*t*_1/2_) of the dark re-reduction of P_700_ after FR illumination in leaves of *A. thaliana* and ephemerals grown under different light conditions.

	*t*_1/2_ (s)
*A. thaliana* LL	1.60 ± 0.14^b,c^
*A. thaliana* HL	0.90 ± 0.09^a^
*A. pumila* LL	1.68 ± 0.09^b,c^
*A. pumila* HL	1.50 ± 0.11^b^
*S. altissimum* LL	1.74 ± 0.08^c^
*S. altissimum* HL	1.51 ± 0.08^b^

*t_1/2_ represents the half-time of dark re-reduction of P_700_ after FR illumination in the presence of DCMU. Data in the table are expressed as means ± SD from three independent experiments. Values marked with different letters were significantly different according to Student’s t-test (P < 0.05)*.

### The Ephemerals Sustained Stable Reaction Center Structure of Both PSs in Long Term Adaptation to the High Light Conditions

**FIGURE 10:**
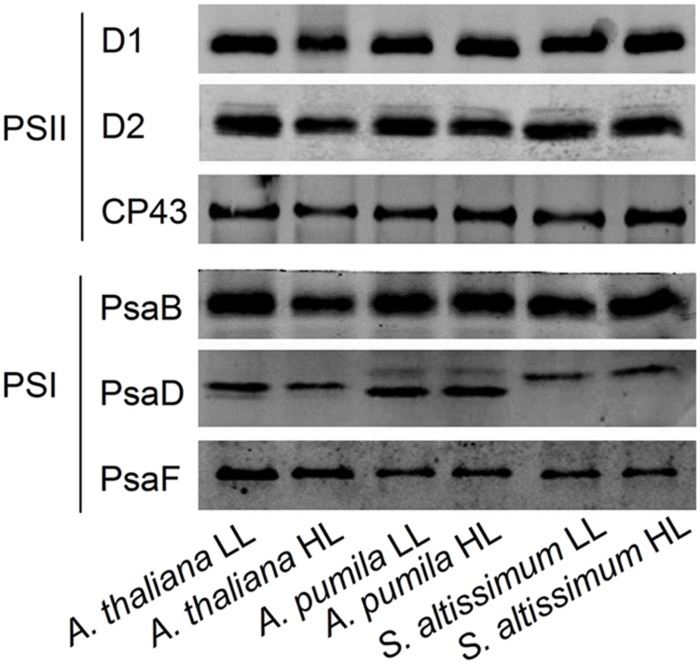
**Immunodetection of thylakoid membrane proteins from ephemerals and *A. thaliana* grown at LL and HL intensities**. Thylakoid membrane proteins (with 1 μg Chl) were separated by SDS-urea-PAGE, and the blots were probed by individual antibodies against D1, D2, CP43, PsaB, PsaD, and PsaF.

The changes in the structure of the two PSs were investigated in *A. thaliana* and ephemerals grown under different light conditions. The levels of the thylakoid membrane protein of different species grown under both the LL- and HL-conditions were performed by western blot analysis using antibodies against various thylakoid membrane proteins (**Figure 10**). It is clearly to see that not only the protein levels of the PSII (D1, D2, and CP43), but also those of PSI (PsaB, PsaD, and PsaF) in the HL-grown *A. thaliana* decreased obviously compared with the LL-grown ones, which was in accordance with the reduced PSII and PSI activities in the HL-grown *A. thaliana*. On the contrast, the ephemerals retained stable PSII and PSI RC under HL conditions, compared with the LL-grown ones. The contents of CP43 increased slightly in the HL-grown *A. pumila* and *S. altissimum*. Furthermore, content of PsaD of *S. altissimum* grown under both light conditions were less than those of *A. thaliana* and *A. pumila*, and contents of PsaF of *A. pumila* and *S. altissimum* were obviously less than that of *A. thaliana*.

## Discussion

Ephemerals have evolved a variety of special characteristics to adapt to the extremely harsh environment in the Gobi area, e.g., the leaves of the ephemerals are able to tolerate high temperature for efficient photosynthesis (Forseth and Ehleringer, 1982a) and capable of osmotic adjustment according to the soil water potentials (Forseth and Ehleringer, 1982b). Highly efficient photosynthesis is one of the most prominent features of the ephemerals, which provides the carbon for them to complete their life cycles within the rainfall season (Yuan et al., 2009). In order to reveal the mechanisms of ephemerals’ high photosynthetic efficiencies under strong light conditions, we investigated the characteristic responses to high irradiance in the three C_3_ species, a spring ephemeral *A. pumila* with close phylogenetic relationship to the reference plant *A. thaliana* and a second spring ephemeral *S. altissimum* widely spread in the Gobi district and a typical habitant in drought regions with high irradiances (Allen and Knight, 1984). Those three species were chosen for the study based on the premise that the mechanisms can be highlighted only when plants with similar life spans and morphologies defining light capture efficiency are compared. *A. pumila*, also named *Olimarabidopsis pumila* (*O. pumila*) by Al-Shehbaz et al. (1999), is a phylogenetic relative to *A. thaliana*. *S. altissimum* is a species of the genus *Sisymbrium of Sisymbrieae*, also belonging to the Cruciferae. Both *A. pumila* and *S. altissimum* are spring ephemeral species widely distributed in the Gobi district in the southern margin of the Gurbantunggut Desert and can successfully survive in this harsh environment. *A. pumila* is the closest phylogenetic relative of *A. thaliana* and shares the same habitat with *S. altissimum* (Zhao et al., 2010), therefore, it was chosen as the bridge between *S. altissimum* and the model plant *A. thaliana*, to support the analysis between the phenotypes derived from different genotypes or derived from long-term adaptation to different environments.

### Coordinated Interaction between PSII and PSI Plays an Important Role in Ephemerals for Sustaining Highly Efficient Light Reaction under High Light Conditions

The most dominant feature of the Gobi district is the strong irradiance and high fluctuation in both the quantity and quality of incident solar energy, accompanied by drought and changing temperatures. As one of the habitants in the Gobi region, the ephemerals have always been encountering significant challenges in this unique natural environment. Consequently, it is imperative for them to develop photosynthetic control mechanisms to convert enough solar energy to ATP and NADPH in a precise ratio for carbon fixation on the one hand, and to protect themselves from dangerous over-excitation on the other hand. Plants have evolved a variety of mechanisms to acclimate to the changing irradiances at various organizational levels from the whole organism to cellular and/or molecular levels (Koller, 1990; Anderson et al., 1995; Murchie and Horton, 1997; Horton et al., 2005; Foyer et al., 2012; Ruban et al., 2012; Cazzaniga et al., 2013; Wilhelm et al., 2014). The sophisticated lateral segregation of the two PSs that separate the “slow reaction in PSII” from the “fast reaction in PSI” in photosynthetic thylakoid membrane provides the basis for the complex photosynthetic control (Trissl and Wilhelm, 1993). The structural and functional relationship between the two PSs plays important roles in regulating photosynthetic electron transport and energy utilization in thylakoid membrane (Pfannschmidt and Yang, 2012). The adjustment of the two PSs activities in thylakoid membrane is an important response to the environment for a fluent electron transport to ensure the best photosynthetic performance (Chow et al., 1990; Melis et al., 1996; Walters, 2005; Pfannschmidt and Yang, 2012). One striking characteristics of ephemerals is that the relationship of PSII/PSI activities is very stable under different light conditions in spite of the significant variations in photochemical activities in the two individual PSs, which should be attributed to the regulated kinetics of D1 protein synthesis. Changing from LL to HL, the photochemical efficiency of PSII and the electron transport rate increased significantly without leading to an increased excitation pressure of PSII in the two ephemerals (**Figure 3**). The markedly increased photochemical activity in PSII, as indicated by the accelerated I-P rise in OJIP kinetic curves (**Figures 5B,C**), was accompanied by the simultaneously increased PSI activity, measured as the increased 820 nm absorption (**Figures 8E,F**). In addition, the unchanged ratio of Y(II)/Y(I) in the two ephemerals under both light conditions also indicated a balanced distribution of quantum yield between the two PSs even under HL conditions (**Figure 9B**). It is clear to see that the ephemerals are able to coordinate PSII and PSI functions and provide ideal conditions for high efficient photochemical reactions via regulation of the photosynthetic control upon the electron transport routes at several important steps.

The mechanisms of the ephemerals sustaining stable relationship of the PSII and the PSI activities resulted from long-term adaptation to the rigorous variations in the Gobi region. On the one hand, they triggered several regulatory processes increasing LEF enormously (**Figure 3**), accompanied by only a slight increase in the NPQ and CEF (**Figures 6**, **8G–I** and **9C**), to avoid the enhancement of the excitation pressure and the further photodamage to PSII. On the other hand, D1 protein contents were much higher in the ephemerals, either via accelerated D1 protein synthesis (*A. pumila*) or via slow degradation of D1 protein against high irradiance (*S. altissimum*; **Figure 7**), which resulted in stable electron transport from PSII to PSI. The low excitation pressure in HL-grown *A. pumila* and *S. altissimum* promoted the charge separation and drove the subsequent electron transport through PSII (**Figure 3**). Further analysis indicated that the enhanced electron transport downstream of Q_A_, viewed via the enhanced electron transport from Q_A_ to Q_B_, from Q_B_ to PQ (**Figures 5A–C**, Strasser et al., 1995; Hill et al., 2004), and from PQ to P_700_ (**Figures 8A–F**), played key roles in regulating the electron transport between the two PSs. In addition, the PSI activities in the ephemerals increased from the view of the increased oxidable P_700_ (**Figure 8**) and the enhanced Y(I) (**Figure 9A**). In conclusion, the redox poise in the thylakoid membrane of ephemerals was sustained even under HL conditions, which in turn, maintained the stable electron transport rate. It is clear that the primary reaction and electron transport in thylakoid membranes is significantly enhanced, which gives rise to the question of what is the relationship between the enhanced electron transport in thylakoid membrane and the carboxylation reaction in the stroma. Further research is needed to elucidate the influence of carboxylation process on the regulatory light reaction processes under HL conditions.

### Elevated Linear Electron Transport in the Ephemerals under High Light Conditions is based on the Stable Photosystem II Supercomplexes

Photosynthetic organisms have developed extensive capacities to respond to variations in light intensities at different dynamics (Kanervo et al., 2005; Takahashi and Badger, 2011). NPQ is a main short-term response to strong light whereby plants dissipate excessive excitation harmlessly to protect PSII RCs from photoinhibition (Lambrev et al., 2012; Ruban et al., 2012). NPQ can be analyzed in terms of three components (energy dependent quenching, that is qE, qT, and qI) on the basis of relaxation kinetic differences (Müller et al., 2001). Our results clearly demonstrated that part of the marked increase in NPQ of the HL-grown *A. thaliana* was originated from the significantly increased photoinhibitory quenching, qI, the slowest component relaxing in a range of hours (**Figure 6**). Besides the quick response in NPQ, plants also possess long-term regulations that result in altered levels in gene expression and regulated PS stoichiometry. It is reasonable to propose that some adaptive/protective mechanisms for PSII may be triggered or up-regulated in ephemerals during long-term evolution, so that the contents of PSII core subunits of ephemerals were able to remain highly stable and the PSII-LHCII supercomplexes also remain unchanged under HL conditions (**Figure 10**). To a certain extent, this structural stability provides a structural basis to sustain efficient electron transfer, and as a result, to utilize more excitation energy for photochemical reaction and to maintain low level of photoinhibition.

Photoinhibition is a state of physiological stress in oxygenic photosynthetic organisms exposed to excess light resulted from the imbalance between light absorption and carbon assimilation. It leads to a reversible and irreversible inactivation of electron transport, and downregulation of the PSII activity (Aro et al., 1993; Andersson and Barber, 1996; Andersson and Aro, 2001; Murata et al., 2007). The D1 protein of PSII is the primary target of photodamage, and therefore, it undergoes constantly degradation and *de novo* synthesis (Aro et al., 1993; Andersson and Aro, 2001). The observation revealed that D1 protein contents in the ephemerals were very stable during high irradiance treatment when remarkable D1 protein degradation already occurred in *A. thaliana* (**Figure 7F**), which demonstrated that the sustained PSII supercomplexes were the basis for the enhanced linear electron transport.

High-light plants and sun leaves possess lower amounts of LHCs, but high levels of the photosynthetic electron transport chain components, which makes them possess functional characteristics of the higher rate of electron transport, the powerful capacity of NPQ, and the efficient D1 protein turnover (Park et al., 1997; Zulfugarov et al., 2007). As high-light adaptive plants, ephemerals not only share the traits with other sun plants, but also possess their own particularities in acclimating to the high irradiance. One of the prominent traits of the ephemerals is the ability to adjust the photochemical activities in different PSs to favor the stable ratio of PSII/PSI activities, which is an important factor for the high photosynthetic efficiency in the ephemerals. Previous studies have shown that D1 degradation in sun plants are higher than that in shade plants (Öquist et al., 1992b), because the higher D1 degradation is not only a critical step of an active PSII repair cycle, but also an effective process protecting PSI from irreversible damage (Andersson and Aro, 2001; Tikkanen et al., 2014). D1 protein degradation and repair are the two key steps regulating photoinhibition by different mechanisms (Edelman and Mattoo, 2008). The dynamic analysis on D1 protein contents during photoinhibitory process of the ephemerals revealed that the two ephemerals sustained the D1 protein contents through different mechanisms (**Figure 7**). *A. pumila* retained via highly effective D1 protein synthesis, probably related to an altered regulation of PSII turnover process, while *S. altissimum* sustained D1 protein content with an increased resistance to D1 protein degradation, which might be related to both the stable PSII/PSI activities with fluent LEF and long-term adaptation of the D1 protein turnover. Further research is needed to illuminate the different mechanisms for sustaining PSII supercomplexes in the different ephemerals.

## Conclusion

The presented results revealed that high photosynthetic efficiency in ephemerals under strong light conditions was attributed to the improved coordination between the two PSs. Under strong light conditions, ephemerals have the ability to adjust the photochemical activities of both PSs to ensure an unimpeded LEF and the structural stability of PSII. The sustaining of stable PSII RCs of ephemerals under strong light conditions provides the structure basis for a highly efficient electron transfer and photosynthetic yield. We suggest that the increased stability of the PSII structure in ephemerals is the results of long-term adaptation of the ephemerals to the strong light conditions.

## Author Contributions

WT and CY designed the experiments. WT and YL performed the research and data analysis. WL, LW, XX, YZ, and CW took part in the data analysis and discussion. WT and CY wrote the paper.

## Conflict of Interest Statement

The authors declare that the research was conducted in the absence of any commercial or financial relationships that could be construed as a potential conflict of interest.

## ABBREVIATIONS

*A. pumila*: *Arabidopsis pumila*
*A. thaliana*: *Arabidopsis thaliana*
AL: actinic light
CEF: cyclic electron flow
Chl: chlorophyll
*E*_k_: saturation index of photosynthetic capacity
*F*_0_: the minimum fluorescence intensity in the dark-adapted state
*F*_m_: the maximum fluorescence intensity in the dark-adapted state
*F*_m_′: the maximum chlorophyll fluorescence intensity of leaves exposed to actinic light
*F*_t_: the steady state fluorescence intensity during illumination
*F*_v_: variable fluorescence
*F*_v_/*F*_m_: maximal photochemical efficiency of PSII
LL: low light
HL: high light
ML: measuring light
NPQ: non-photochemical quenching
PAM: pulse amplitude modulation fluorometer
*P*_m_: the maximum P_700_^+^ signal
*P*_m_′: maximum P_700_^+^ signal under the actinic light
PQ: plastoquinone
PSI: photosystem I
PSII: photosystem II
Q_A_: primary electron acceptor in PSII
Q_B_: secondary electron acceptor in PSII
qP: the coefficient of photochemical quenching
RC: reaction center
rETR: relative electron transport rate through PSII
ROS: reactive oxygen species
*S. altissimum*: *Sisymbrium altissimum*
*S*_m_: complementary area above the OJIP transient with the maximum measured fluorescence intensity as upper boundary normalized to *F*_v_
*T*_m_: the illumination time needed to reach the maximum fluorescence intensity
Y(CEF): quantum yield of cyclic electron flow
Y(I): quantum yield of PSI
Y(II): quantum yield of PSII.
