# Supplementary material for: Spring Ephemerals Adapt to Extremely High Light Conditions via an Unusual Stabilization of Photosystem II
Source: Front Plant Sci. 2016 Jan 6;6:1189. doi: 10.3389/fpls.2015.01189 (PMC4702278; doi:10.3389/fpls.2015.01189)
Supplement: Supplementary file 1 [file Presentation_1.PDF]

Spring ephemerals adapt to extremely high light conditions ~~by~~ an unusual stabilization of photosystem II

Wenfeng Tu[a], Yang Li[a], Wu Liu[a], Lishuan Wu[a], Xiaoyan Xie[a], Yuanming Zhang[b], Christian Wilhelm[c], Chunhong Yang[a,*]

[a] Key Laboratory of Photobiology, Institute of Botany, Chinese Academy of Sciences, Beijing, China

[b] Key Laboratory of Biogeography and Bioresource, Xinjiang Institute of Ecology and Geography, Chinese Academy of Sciences, Urumqi, Xinjiang, China

[c] Institute of Biology, Department of Plant Physiology, University of Leipzig, Johannisallee 21-23, 04103, Leipzig, Germany

* Correspondence:

Dr. Chunhong Yang, Key Laboratory of Photobiology, Institute of Botany, Chinese Academy of Sciences, Nanxincun 20, Xiangshan, Beijing 100093, China

e-mail: yangch@ibcas.ac.cn.

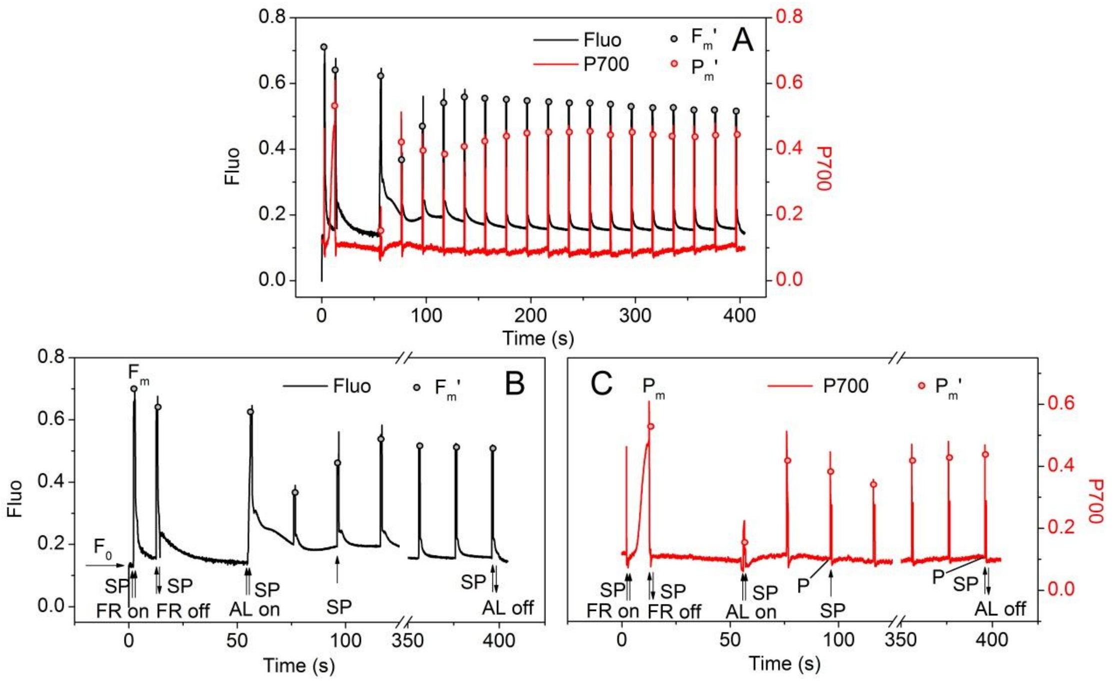

Supplementary Figure 1 Simultaneous measurements of fluorescence and P700 signals using a Dual-PAM-100 measuring system. (A) Simultaneous recording of slow induction curves of fluorescence and P700 with saturating pulse. **(B)** Measurement of fluorescence in detail. **(C)** Measurement of P700 in detail. F_0, The minimal fluorescence measured in the dark-adaptation state with a weak measuring

light. SP, saturating pulse. F_m, the maximum fluorescence detected by applying an SP. FR, far red light. P_m, the maximal P_{700} change determined through application of an SP after FR pre-illumination for 10 s. AL, actinic light. F_m', the maximum fluorescence signal determined by applying an SP under AL. P_m', the maximum P_{700}^+ signal determined by applying an SP under AL without FR pre-illumination. P, The P_{700}^+ signal recorded just before an SP, which was applied to determine F_m' and P_m'.
